# Analysis of Intracellular State Based on Controlled 3D Nanostructures Mediated Surface Enhanced Raman Scattering

**DOI:** 10.1371/journal.pone.0015836

**Published:** 2011-02-24

**Authors:** Waleed Ahmed El-Said, Tae-Hyung Kim, Hyuncheol Kim, Jeong-Woo Choi

**Affiliations:** 1 Interdisciplinary Program of Integrated Biotechnology, Sogang University, Seoul, Republic of Korea; 2 Department of Chemical and Biomolecular Engineering, Sogang University, Seoul, Republic of Korea; Texas A& M University, United States of America

## Abstract

Near-infrared surface-enhanced Raman spectroscopy (SERS) is a powerful technique for analyzing the chemical composition within a single living cell at unprecedented resolution. However, current SERS methods employing uncontrollable colloidal metal particles or non-uniformly distributed metal particles on a substrate as SERS-active sites show relatively low reliability and reproducibility. Here, we report a highly-ordered SERS-active surface that is provided by a gold nano-dots array based on thermal evaporation of gold onto an ITO surface through a nanoporous alumina mask. This new combined technique showed a broader distribution of hot spots and a higher signal-to-noise ratio than current SERS techniques due to the highly reproducible and uniform geometrical structures over a large area. This SERS-active surface was applied as cell culture system to study living cells *in situ* within their culture environment without any external preparation processes. We applied this newly developed method to cell-based research to differentiate cell lines, cells at different cell cycle stages, and live/dead cells. The enhanced Raman signals achieved from each cell, which represent the changes in biochemical compositions, enabled differentiation of each state and the conditions of the cells. This SERS technique employing a tightly controlled nanostructure array can potentially be applied to single cell analysis, early cancer diagnosis and cell physiology research.

## Introduction

In recent years, extensive efforts have been made to develop a method of monitoring the behavior of proteins and other macromolecules inside a cell during the key cellular processes such as cell differentiation, division, and apoptosis. Current techniques have enabled single-molecule imaging that allows intensive analysis of the biochemical composition inside the cells [1]. However, most of these techniques require complex and time-consuming steps such as cell fixation, lysis, extraction or the introduction of molecular probes. Additional labeling techniques also require cumbersome protocols. These steps often make the techniques more costly and can cause non-specific sample binding, which increases the possibility of false-positives.

Optical and spectroscopic methods such as spectrophotometric methods [2], fluorescence microscopy [3] and confocal microscopy [4] can be utilized for the analysis of living cells, but have critical disadvantages due to the presence of exogenous fluorophores, which results in their providing limited information specific to only a small range of subcellular components. Non-optical methods such as cyclic voltammetry (CV) or differential pulsed voltammetry (DPV) have also been employed to analyze the cellular behavior without fluorescence dyes [5], [6]; however, the voltammetric signals achieved using electrochemical tools only represent cell viability, which is insufficient for intensive cell-based research.

Raman spectroscopy is a powerful analytical technique for the analysis of living cells that is rapid, reagent-free, and non-destructive [7]. However, biomedical application of Raman spectroscopy has been limited because it produces weak and unstable signals.

SERS phenomena offer a method of overcoming the critical limitations of Raman spectroscopy (low sensitivity) via a 10^9-15^ fold increase in Raman sensitivity [8], [9]. Several strategies have been reported to obtain SERS signals such as immobilization of metal colloids and metal particles on a plate. A SERS-active surface that uses a non-uniform distribution of gold (Au) nanoparticles (NPs) on a 3-aminopropyltrimethoxysilane (APTMS) functionalized ITO substrate has been reported [7]. However, any small variation in the local arrangement of nanostructures (patterns/shapes) used as SERS-active substrates leads to critical changes in the SERS signals due to the high sensitivity of the hot spots. In addition, the surfactant involved in the deposition step of metal NPs and the organic linkers (e.g. APTMS) reduces the enhancing effects and interferes with the SERS signals of the target molecules [10], [11]. We also previously reported a simple method of enhancing the Raman signal by fabricating Au nanoflower modified ITO substrates to detect changes in cell behavior after treatment with chemotherapeutic agents. This substrate demonstrated highly enhanced Raman signals [12]; however, the size and shape of the Au nanoflowers structures that was not uniform enough that could be affected on the distribution of enhanced factor, which can induce differently enhanced Raman signals. Accordingly, a geometrically well organized clean SERS-active substrate that allows control of both the size and shape of the nanostructures is highly desirable [13]–[16].

Conversely, the metal particles dispersed in a colloidal suspension have been employed to induce a SERS effect for the detection of Raman signals from living cells under physiological-like conditions [17], [18]. However, localization of colloidal particles inside a living cell was found to be difficult to control and to cause the aggregation of non-homogeneous particles, which dramatically decreases the efficiency of Raman signal enhancement from one point to another within the cell surface. Furthermore, antibody-conjugated metal particles have also been evaluated as SERS-active agents to overcome the aforementioned limitations; however, the antibody was reported to cause unwanted SERS signals that could not easily be distinguished from Raman signals originating from target molecules inside a cell [18].

Here, we report the combined SERS analysis tool with a uniformly distributed Au nano-dots array on an ITO surface, which provides low signal variances of SERS signals with high intensity and reproducibility. To determine the superiority of Au nano-dot substrate mediated SERS effects, the peak intensities of *p*-aminothiophenol (PATP) from an Au nano-dot array were initially compared with those from a non-homogeneous SERS-active substrate. This non-homogeneous SERS-active substrate was fabricated by the immobilization of Au NPs, which have approximately equal numbers of Au nano-dots distributed over an area with the same size as that of the nano-dot array. Thereafter, we used this SERS-active substrate in several applications, especially for cell-based research. This transparent SERS-active Au nano-dots modified allowed us to study living cells *in situ* within their culture environment without any external preparation processes. Specifically, we evaluated its use for distinguishing different cell lines (MCF-7, HeLa and HepG2), cells at different stages of the cell cycle (G_1_/G_0_ and S/G_2_ phase), live/dead cells and even for differentiation of biochemical compositions of single cells at different spots. The developed technique was also used to distinguish the normal breast cell (HMEC) and breast cancer cell (MCF-7) line to demonstrate its capacity as a label-free cancer diagnostic tool.

## Results

### Distribution and variance of the SERS enhancement from different SERS Substrates at each spot using the AFM-Raman conjugated technique

**Figure 1:**
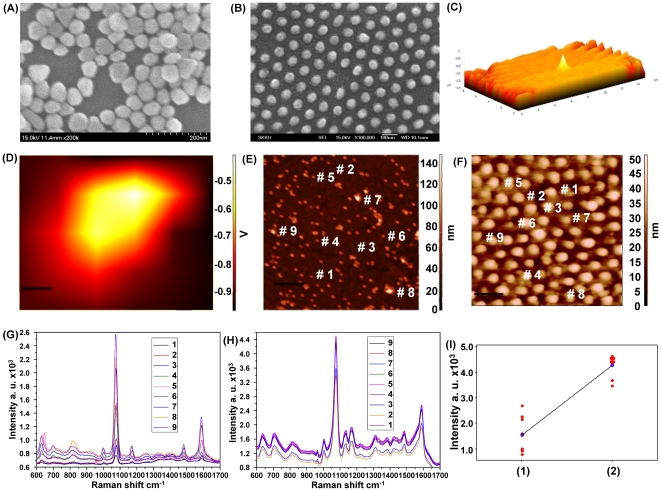
Characterization of the SERS-active substrates. (**A**) SEM topography of Au NPs deposited on the APTMS/ITO substrate. (**B**) SEM topography of the Au nanodot array fabricated on an ITO surface through an Al mask. (**C**) Three-dimensional confocal image of the AFM tip. (**D**) Confocal image of the AFM tip focused at the same point as the IR laser. (**E**) AFM topography of the PATP/Au NPs deposited on the APTMS/ITO substrate. (**F**) AFM topography of the PATP/Au nanodot array fabricated on an ITO surface. (**G**) SERS spectra of PATP at several points from the Au NP array substrate. (**H**) SERS spectra of PATP at several points from the Au nanodot array substrate. (**I**) Intensity distributions of the Raman peak corresponding to NH group within the SERS intensity peaks recorded from the PATP monolayer immobilized on the (**1**) Au NP array and (**2**) Au nanodot array.

**Figure 2:**
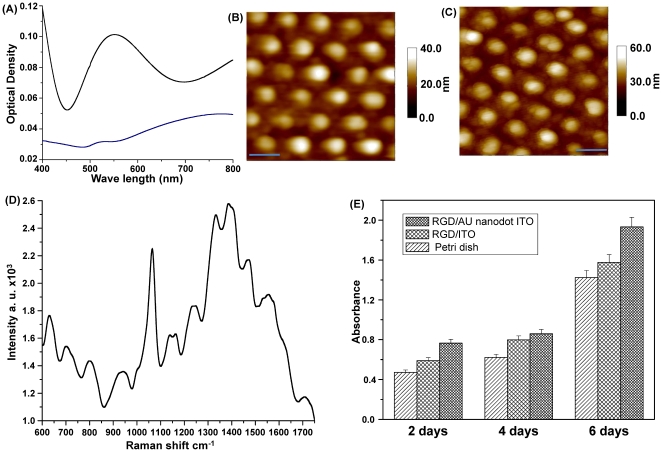
Validation of the substrate as a cell-based chip. (**A**) Surface plasmon absorption spectra of the Au NP array (blue line) and the Au nanodot array (black line). (**B**) AFM image of the Au nanodot/ITO substrate. (**C**) AFM image of RGD-peptide immobilized on the Au nanodot/ITO substrate. Scale bar: 100 nm. (**D**) Raman spectrum of an RGD-peptide immobilized on the Au nanodot/ITO. (**E**) Proliferation rate of HMEC cells determined *via* an MTT assay as a function of absorbance until day 6.

We constructed two different substrates containing approximately equal numbers of Au nanostructures that contained SERS-active spots that were distributed homogeneously and non-homogeneously to compare the SERS signal variances and intensities. The non-homogeneous SERS–active substrate was fabricated by self-assembly of 60 nm Au NPs on an APTMS-functionalized ITO surface (**Figure 1A**). Conversely, the homogeneous nanostructure arrays composed of 60 nm Au nano-dots were prepared by thermal evaporation of pure Au metal on an ITO surface through a nanoporous alumina mask (**Figure 1B**). Next, the Raman spectra of PATP immobilized on both substrates were determined at various spots. The optimal position of the laser beam on the nanostructured substrates was coordinated by matching the Raman laser focal spot with the top of the atomic force microscopy (AFM) tip (**Figures 1C and 1D**), which allowed us to correlate the Raman signal with the AFM topographic image (**Figures 1E and 1F**). As shown in **Figure 1G**, the Raman spectra of the PATP/Au NPs array from nine different points demonstrate a large variance in Raman enhanced signals due to randomly distributed SERS–active hot spots. Since SERS signals were enhanced at spots in which NPs aggregated, the spots that contained few Au NPs provided weak Raman intensities. In contrast, the Raman signals of the PATP/Au nanodot array from nine different spots that were selected at random (**Figure 1H**) showed a significantly enhanced Raman shift with very low fluctuation. Moreover, the average intensities of the Raman shift at 1077 cm^−1^ (–NH group) from the non–homogeneous and homogeneous SERS–active substrates (**Figure 1I**) differed significantly at 1531 and 4285 cm^−1^, respectively. These data indicate that the Au nano–dot array was superior to the non-homogenous Au NPs array with respect to signal strength, reproducibility, and variation. Moreover, the Au nano–dot array showed a stronger surface Plasmon absorption band than that of the non–homogeneous Au NP array, possibly due to the hot spots being homogenously distributed over the surface (**Figure 2A**).

### SERS signal enhancement from living cells

The cell nucleus contains most of a cell's genetic information and is composed of DNA molecules and numerous proteins (its own structural material). Hence, SERS has been used to investigate the structure-function relationship in the cell nucleus. However, SERS–based investigation of a cell nucleus is challenging due to the existence of several cellular barriers that limit the delivery of SERS–active colloidal NPs to the cell nucleus [19]. Moreover, Au NP–targeting of cancer cell nuclei has been found to influence cellular function, causing cytokinesis arrest, DNA damage, and programmed cell death, which leads to failed cell division, thereby resulting in apoptosis [20]. Hence, our developed uniformly–distributed, Au nano–dot substrate was used for SERS–based investigation of the cell nucleus and cytoplasm and was found to be able to overcome the limitations of colloidal NPs.

Before cell immobilization on the different substrates, RGD (Arg–Gly–Asp) peptide with the cysteine (Cys) residue was self-assembled on the Au nanostructures arrays to enhance cell adhesion. The immobilization level of the RGD peptide on the Au nano-dot array was confirmed by AFM analysis (**Figures 2B and 2C**) and Raman spectroscopy. The SERS spectra of the RGD peptide (**Figure 2D**) were characterized by peaks [21] at 1700 cm^−1^ (carbonyl group of aspartic acid), 1455 cm^−1^ (C–H bend), 1390 cm^−1^ (str._sym._ COO^–^), 1240 cm^−1^ (amide III), 940 cm^−1^ (str. C–COO^–^), 630 cm^−1^ (wagging COO^–^), and approximately 1060 and 1550 cm^−1^ (rocking NH_2_). MTT assay was conducted to compare the biocompatibility of the RGD peptide coated ITO substrate, RGD/Au nano–dot, and cell culture plate. The RGD-coated Au nano-dot substrate showed the highest cell proliferation rate (**Figure 2E**).

To determine if the Au nano-dot substrate was superior to the non-homogenous Au NPs array and colloidal Au NPs for use as an SERS-active substrate, HEK 293T cells were immobilized on a non–homogeneous Au NPs array and a homogeneous Au nano–dot array or were incubated with a colloidal Au NPs solution on a glass cover-slip.

**Figure 3:**
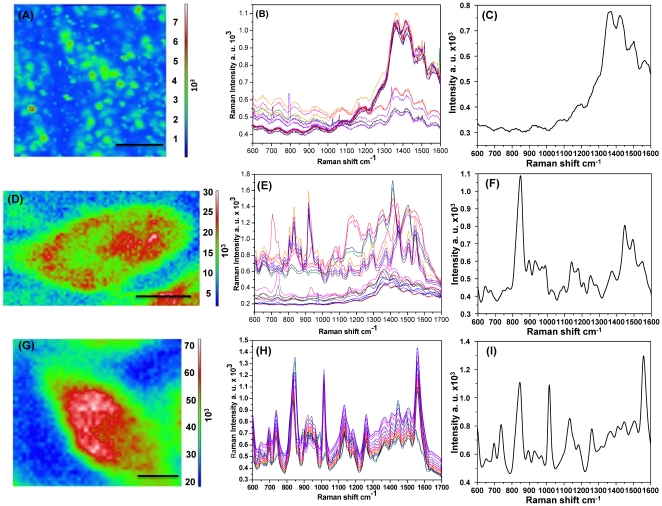
SERS mapping image and spectra of HEK 293T cells. (**A**) SERS map image of cells incubated with colloidal Au NPs. (**B**) SERS spectra from twenty different points inside the cell nucleus incubated with colloidal Au NPs. (**C**) Mean SERS spectrum of twenty spectra at different points inside the cell nucleus incubated with colloidal Au NPs. (**D**) SERS map image of cells immobilized on the randomly distributed Au NP array. (**E**) SERS spectra from twenty different points inside the cell nucleus immobilized on the Au NP array. (**F**) Mean SERS spectra of twenty different points inside the cell nucleus immobilized on the Au NP array. (**G**) SERS map image of cells immobilized on the homogenous Au nanodot array. (**H**) SERS spectra of twenty different points inside the cell nucleus immobilized on the Au nano-dot array. (**I**) Mean SERS spectra of twenty different points inside the cell nucleus immobilized on the Au nano-dot array. Scale bar: 10 µm.

SERS mapping was then conducted for each cell (**Figures 3A, 3D, and 3G**), which enabled the SERS spectra to be obtained at each point within the map images. As shown in **Figure 3A**, the SERS map image of cells incubated with the colloidal Au NPs solution revealed unclear or no cell image due to the limited delivery of Au NPs into cells. However, the SERS map image of cells immobilized on a non–homogeneous Au NPs substrate produced a better image than that of the colloidal Au NP solution (**Figure 3D**). Conversely, cells on the Au nano–dot substrate (**Figure 3G**) yielded a high resolution SERS map image, which clearly differentiated the cytoplasm and nucleus of the cells.

Raman spectra were also obtained and analyzed from 20 different points inside the cell nucleus to confirm the superiority of the Au nano–dot substrate (**Figures 3B, 3E, and 3H**). The Raman spectra obtained from the colloidal Au NP solution showed weak and noisy mean spectra (**Figures 3B and 3C**) due to a large gap between the Au NPs and the cellular components. Although the randomly distributed Au NP array showed more enhanced Raman effects than the colloidal Au NP solution, the signals remained unreliable (**Figures 3E and 3F**). The SERS spectra obtained from a cell on the homogeneously distributed Au nano–dot array demonstrated clear spectra, even with a slight shift and variation in some bands (**Figures 3H and 3I**), which might have been due to their heterogeneous cellular composition.

The intensities of three different Raman bands at 835, 1006, and 1155 cm^−1^ were recorded from 20 different points within the nucleus of cells that were immobilized on non-homogeneous and homogeneous substrates. These data were statistically analyzed using a T–test (**Figure S1**), which revealed low p–values of approximately 0.007, 0.000, and 0.004, respectively (**Table S1A–C**), indicating low signal variations in the nano–dot array. Based on these data, the Au nano–dot array is a more efficient SERS-active substrate than the colloidal Au NP solution and non-homogeneously distributed Au NP array.

### Differentiation of different cancer cell lines

**Figure 4:**
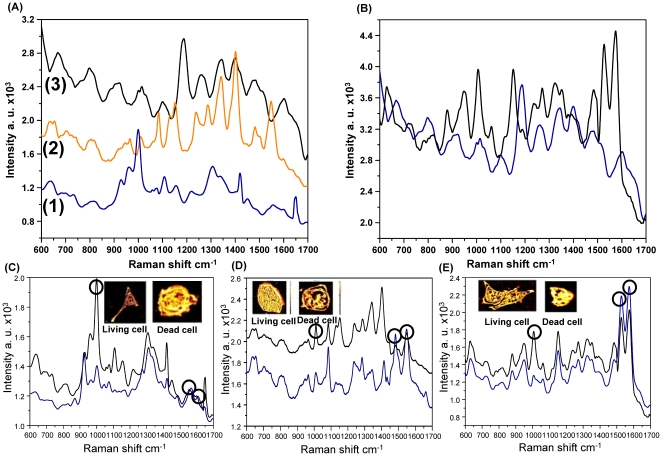
SERS spectra of different cell lines. (**A**) SERS spectra of (**1**) HeLa, (**2**) HepG2, and (**3**) MCF-7 cells. (**B**) SERS spectra of (blue) MCF-7 and (black) HMEC cells. (**C**) SERS spectra of living HeLa cells (black curve) and dead HeLa cells (blue curve). Inset contains the confocal microscopic images of living and dead HeLa cells. (**D**) SERS spectra of living HepG2 cells (black curve) and dead HepG2 cells (blue curve). Inset contains the confocal microscopic images of living and dead HepG2 cells. (**E**) SERS spectra of living HMEC cells (black curve) and dead HMEC cells (blue curve). Inset contains the confocal microscopic images of living and dead HMEC cells. Circles in **Figs. C, D, and E** indicate the primary differences between the SERS spectra of living and dead cells.

**Figure 4A** shows SERS spectra from three different cancer cell lines (HeLa, HepG2, and MCF-7) that produce different spectra due to inherent molecular differences [22]. The MCF-7 cell line contains a relatively low level of nuclear material and unordered proteins when compared to the two other cell lines, which is indicated by changes in the intensity of peaks at 721, 783, 1305, 1381, and 1450 cm^−1^, as well as those of the DNA backbone (PO_2_) (827 and 1095 cm^−1^), and of unordered proteins (1250 and 1655 cm^−1^). The changes in the Raman spectra at several Raman shifts also represent varying amounts of α-helix proteins (935, 1265, and 1655 cm^−1^), phospholipids (721, 1095, 1125, and 1335 cm^−1^), and lipids (1095 and 1305 cm^−1^). Several Raman peaks at 1611, 1212, 1153, and 959 cm^−1^ were observed from the HeLa and MCF–7 cell lines. However, for the MCF–7 cell line, the peak intensities of the Raman shift were much weaker than those from HeLa cells. Raman peaks at 783, 721, and 673 cm^−1^ were detected from MCF–7 and HepG2 cells, but not from HeLa cells. Moreover, differences in the intensity of Raman peaks representing the amide III, CH_2_, and other chemical bands were observed (**Tables S2, S3, S4**). Therefore, our substrate was able to differentiate cancer cell lines successfully.

### Differentiation of normal and cancer cells derived from the same organ

The SERS spectra from breast normal (HMEC) and breast cancer (MCF-7) cells shown in **Figure 4B** exhibited different Raman shifts (**Tables S4 and S5**) and Raman relative peak intensities. The Raman spectra of cancer cells showed relatively low peak intensities at 865–895, 1003, and 1070 cm^−1^, but relatively high peak intensities at 1425 and 1657 cm^–1^ when compared to those of normal cells, indicating structural changes and down or up–regulation of biomolecules in cancer cells. The Raman peaks at 1003 and 1210 cm^−1^, assigned to Phe and Trp, demonstrated lower intensity values and fewer Phe and Trp amino acid components in normal cells (HMEC) than in MCF-7 cancer cells [23], [24]. Slight shifts toward longer wavelengths were observed at 1150 cm^−1^, 1300–1400 cm^−1^, and 1450–1550 cm^−1^, which were attributed to amide I and III bands, CH_3_CH_2_ twisting of proteins/nucleic acids, and C = C stretches in phospholipids in the cancer cells, respectively. These findings indicate that there is an increased percentage of the total Raman-active biomolecular components in MCF-7 cancer cells relative to normal cells. Moreover, a shoulder band at 1650 cm^−1^, which represents an amide I, β-pleated sheet and/or random coil conformation, was observed. These results demonstrated that a cancer cell might also express high concentrations of proteins containing β-pleated sheet structures [24], [25], [26], which induce more mitotic activity than normal cells [27]. These Raman spectral in vitro studies are consistent with previous histopathologic studies of malignancy grading [24], [28].

### Differentiation of live and dead cells

Raman spectra of living and dead cells from three different cell lines (HMEC, HepG2, and HeLa) were obtained and compared to identify the spectral differences (**Figures 4C-E**). Raman spectra obtained from the nucleus of dead cells showed a dramatic decrease across most of the spectral region when compared to those of living cells. In dead cells, the Raman peak intensities decreased at 783 and 827 cm^−1^ and at 1003 and 1337 cm^−1^, demonstrating the breakdown of internal phosphodiester bonds in DNA molecules and decreased protein expression levels, respectively. However, the nucleus of the dead cells showed increased Raman peak intensities at 1578 cm^−1^ and new Raman peaks at 1607 or 1114 cm^−1^, which corresponded to Phe and Try, respectively. These results represented the degradation of DNA molecules and the expression of new aromatic amino acid–rich proteins that normally occur during cell apoptosis [29].

### Detection of biochemical composition of cells at different cell cycle stages

**Figure 5:**
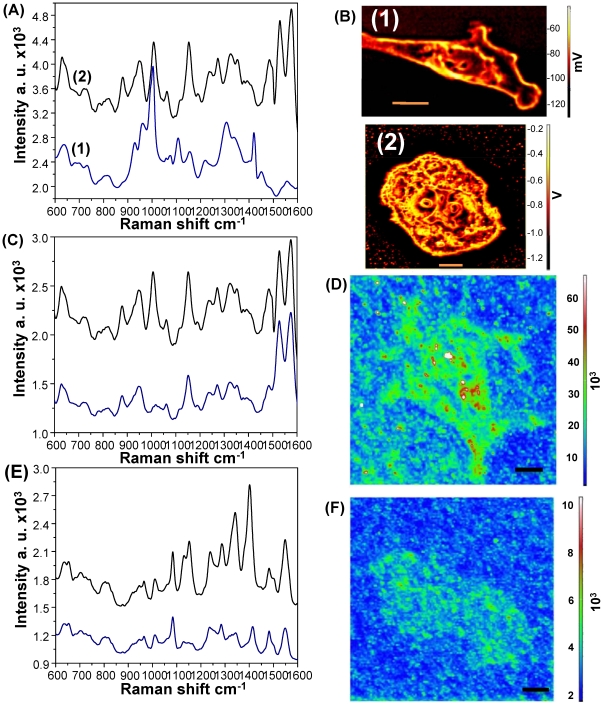
SERS monitoring at different cell cycle stages. (**A**) SERS spectra of (black) resting HMEC cells (G_0_/G_1_) and (blue) mitotic HMEC cells (S/G_2_). (**B**) Confocal images of HMEC cells in the (**1**) resting state (G_0_/G_1_) and (**2**) mitotic state (S/G_2_), (**C**) SERS spectra of HMEC cells from cytoplasm (blue curve) and the nucleus (black curve), and (**D**) SERS map image of HMEC cells. (**E**) SERS spectra of HepG2 cells from cytoplasm (blue curve) and the nucleus (black curve), and (**F**) SERS map image of HepG2 cells. Scale bar: 10 µm.

**Figure 5B** shows the confocal images of cells in the resting (G_1_/G_0_ phase) and mitotic (S/G_2_ phase) stages. Raman spectra from cells in each stage were found to differ significantly (**Figure 5A**). The Raman spectra from the mitotic cells showed increased peak intensities at 783, 1095, and 1578 cm^−1^, suggesting that the mitotic cells contained more nucleic acids than cells in the resting stage. In addition, a strong Raman peak was detected at 1446 cm^−1^, which can be assigned to the C–H bend, including CH_2_ scissors and CH_3_ degenerate deformations. Furthermore, the mitotic cells showed relatively high Raman peak intensities at the 1190–1385 cm^−1^ spectral region that corresponds to proteins, lipids, and nucleic acids (**Figure 5A**). As shown in **Figure 5A**, the Raman spectral intensity values from the nucleus of a mitotic cell were generally higher than those from the nucleus of resting cells, which demonstrated a higher expression level of activated DNA molecules and the presence of a DNA-histone complex.

### SERS mapping for visualization of the distribution of biochemical components within living cells

Finally, our SERS–active substrate was evaluated to analyze the variations in SERS signals obtained from the different regions of HMEC and HepG2 cells. As shown in **Figure 5D** **and** **Figure 5F**, the SERS maps demonstrate the distribution of the different biomolecules within the HMEC and HepG2 cells, respectively. **Figure 5C** **and** **Figure 5E** show the Raman spectra from two specific points corresponding to the cytoplasm and nucleus of living HMEC and HepG2 cells, respectively. The Raman peak intensities from the nucleus were relatively higher than those from the cytoplasm due to the enrichment of DNA and RNA.

## Discussion

Here, we demonstrated the potential for the use of a simple technique that can control the geometrical distribution of the Au nanostructures over a large surface area of transparent ITO substrate as a SERS-active substrate and explored the biochemical composition of living cells. The uniformly distributed hot spots over a large area contributed to significant enhancement of a Raman shift, with a sufficient sensitivity and reproducibility and relatively very low variations at different locations when compared to those of conventional SERS techniques, which generally use irregular or uncontrolled patterns of metallic particles.

To evaluate the feasibility and practical merits of combined the controlled SERS-active nanostructures with the Raman technique, we applied this system to various aspects of cell-based research. It is well-known that the investigation of intracellular components using the SERS technique is very difficult due to the complexity of Raman signals of living cells being undistinguishable from the enhancement of the Raman shift. Hence, we used our technology as a useful candidate to overcome this complicated problem to explore the complex components of living cells and determine both the protein expression levels and distribution. As expected, strong SERS signal enhancement was achieved through the whole cell area, and a clear SERS mapping image was observed over a single cell in very short times not available in the normal Raman or conventional SERS method. The cell nucleus and cytoplasm were also clearly distinguishable in both the SERS mapping image and SERS spectra, which demonstrates the stability and reliability of our SERS-active substrate.

After confirmation of the sensitivity and stability of the SERS signal from cells based on our developed method, we used this technique as a cancer diagnostic tool. In the cancer clinical field, a short detection time for cancer determination is the most important factor because the biopsy process should involve surgery. The time needed to achieve a sufficient intensity for Raman detection was found to be very short (less than 5s) based on our technique; hence, this technique should be very useful in the cancer diagnostic field. The SERS spectra obtained from different cancer cell lines were clearly distinguishable, as well as cells derived from the same organ (breast cell line). The SERS spectra recorded from cancer cell lines tend to have high protein contents, which is consistent with the findings of earlier studies [27]. In addition, similar spectral differences among Raman signals were observed in living and dead cells due to the differences in the numbers of synthesized DNA, DNA damage, cytoplasmic damage and the reduced cytoplasmic process.

Clear Raman spectra with small variations can be applied to many different types of cell based research. To utilize this feature, the SERS-active substrate as cell-based chip that developed here was also used to detect cell cycle-dependent SERS signals because differentiating cells in different cycles is a difficult and time-consuming process that should be properly characterized for intensive cellular research. Based on our SERS-active substrate as cell-based chip, differences in the SERS spectra of cells were successfully detected and found to correspond to the various stages of the cell cycles. Additionally, mitotic cells (in S or G_2_/M phase) were found to show stronger DNA peaks in their Raman spectra when compared to cells in the rest phase (G_1_/G_0_) due to the DNA synthesis, while they showed a lower level of protein expression. These results indicate that cells in different stages can be easily distinguished using our SERS combined technique without any fluorescence or external dyes, which are essential for optical/fluorescence based techniques.

Based on the confirmed superiority of the SERS method presented here and its wide potential application, this technique is sensitive and strong enough to study living cells *in situ* within their culture environment without any external techniques. Moreover, practical techniques such as AFM and confocal microscopy could be easily incorporated into the system to monitor small chemical changes in the cell that might be a precursor of larger morphological changes more sensitively. In addition, the time-dependent biological reactions of cells to toxic agents, which is crucial for drug development, can be efficiently monitored while reducing the time and cost of drug testing. Therefore, it can be concluded that the cutting edge technology presented here has opened a new method of the Raman technique as an extensive cell-based research tool and practical tool for rapid cancer diagnosis without any preparation processes.

## Materials and Methods

### Cells culture

Human liver carcinoma cell line (HepG2), human epithelial carcinoma cell line (HeLa) and human breast adenocarcinoma cell line (MCF-7) were obtained from the Korean Cell Line Bank (Seoul, Korea). While, human embryonic kidney cells (HEK 293T) was purchased from American Type Culture Collection (ATCC) and primary human mammary epithelial cells (HMEC) was obtained from Lonza (Cat. No. CC-2551).

HepG2, HeLa, HEK 293T cell lines were cultured in DMEM supplemented with 10% heat inactivated fetal bovine serum (FBS; Gibco, Carlsbad, CA, USA) and 1% antibiotics (Gibco). While, MCF-7 and HMEC cell lines were cultured in PRMI supplemented with 10% heat inactivated fetal bovine serum (FBS; Gibco, Carlsbad, CA, USA) and 1% antibiotics (Gibco). All cell lines were maintained under standard cell culture conditions at 37°C in an atmosphere of 5% CO_2_. The medium was changed every two days.

### Preparation of the SERS-active substrate

The Au nano-dot array was fabricated on ITO substrates using an anodic nanoporous alumina membrane as an evaporation mask. The nanoporous alumina mask was prepared by a two-step anodization process [30]. Next, the anodic alumina mask with a 60 nm diameter hole was placed on a 20 mm×10 mm ITO substrate for fabrication of the Au nano-dot array. Au was then deposited onto the ITO substrate through the nanoporous alumina mask using a thermal evaporator. After Au deposition, the alumina mask was removed by immersion in 1 M NaOH for several minutes. A 10 mm×10 mm×10 mm (width × length × height) cell chamber was attached to the Au nano-dot-deposited ITO substrate to measure the Raman spectra of living cells under physiological-like conditions. A thin film of RGD-Map-C peptide was fabricated on the Au nano-dot/ITO surface by incubating the samples in 0.01 mg/mL of RGD-Map-C solution in PBS for approximately 12 h, followed by washing with DI water, and drying with N_2_ gas [31]. The cells were transferred into the chip using new culture medium, after which they were allowed to attach and grow for 48 h prior to measurement. The cells were then washed three times with PBS (pH 7.4) and used for the Raman measurements.

### Characterizations of RGD peptide and cells based on AFM, Confocal microscopy and SERS

NTEGRA spectra (AFM-Raman Spectrometer, NT-MDT, Russia) equipped with a liquid nitrogen-cooled CCD detector and an inverted optical microscope (Olympus IX71) were used to evaluate the pharmacology of Au-nanodots/ITO, RGD-peptide/Au-nanodots/ITO and cells using AFM using a semi-contact mode equipped with an inverted optical microscope under liquid conditions. The maximum scan-range of the system was 100 µm×100 µm. The cantilevers used were type NSG01 and had a typical resonant frequency in the range of 115 to 190 kHz and a force constant of 2.5 to 10 N/m. The scan rate was selected to be 0.5 Hz. In addition, confocal and SERS spectra were recorded, with the maximum scan-range in the XYZ directions being 100 µm×100 µm×6 µm, and the resolution of the spectrometer in the XY plane and Z axis being 200 nm and 500 nm, respectively. SERS spectra, SERS mapping and confocal images were recorded using a NIR laser emitting at 785 nm with an irradiation laser power of 3 mW on the sample plane. Twenty scans of 5 s from 600–1750 cm^−1^ were recorded and the mean data were used. A blank spectrum was acquired prior to each step, which allowed the absorbance to be subsequently measured.

## Supporting Information

Figure S1 Probability distributions of SERS intensity peaks from nuclei of HEK 293T cells immobilized on (**1**) an Au NP array; and (**2**) an Au nano-dot array substrate at wavelength **of** (**a**) 835 cm^−1^. (**b**) 1006 cm^−1^. (**c**) 1155 cm^−1^.
(TIF)

Table S1 Independent Student's two-sample T-test statistical analysis for comparing Raman peak intensities from HEK 293T cell nuclei immobilized on (**V1**) an Au NP array; and (**V2**) an Au nanodot array at wavelengths (**a**) 835 cm^−1^ **.** (**b**) 1006 cm^−1^. (**c**) 1155 cm^−1^.
(TIF)

Table S2 Peak locations for SERS spectra of living HaLa cells.
(TIF)

Table S3 Peak locations for SERS spectra of living HepG2 cells.
(TIF)

Table S4 Peak locations for SERS spectra of living HMCF cells.
(TIF)

Table S5 Peak locations for SERS spectra of living MCF-7 cells.
(TIF)
