# Supplementary figures and images for: Analysis of Intracellular State Based on Controlled 3D Nanostructures Mediated Surface Enhanced Raman Scattering
Source: PLoS One. 2011 Feb 24;6(2):e15836. doi: 10.1371/journal.pone.0015836 (PMC3044723; doi:10.1371/journal.pone.0015836)

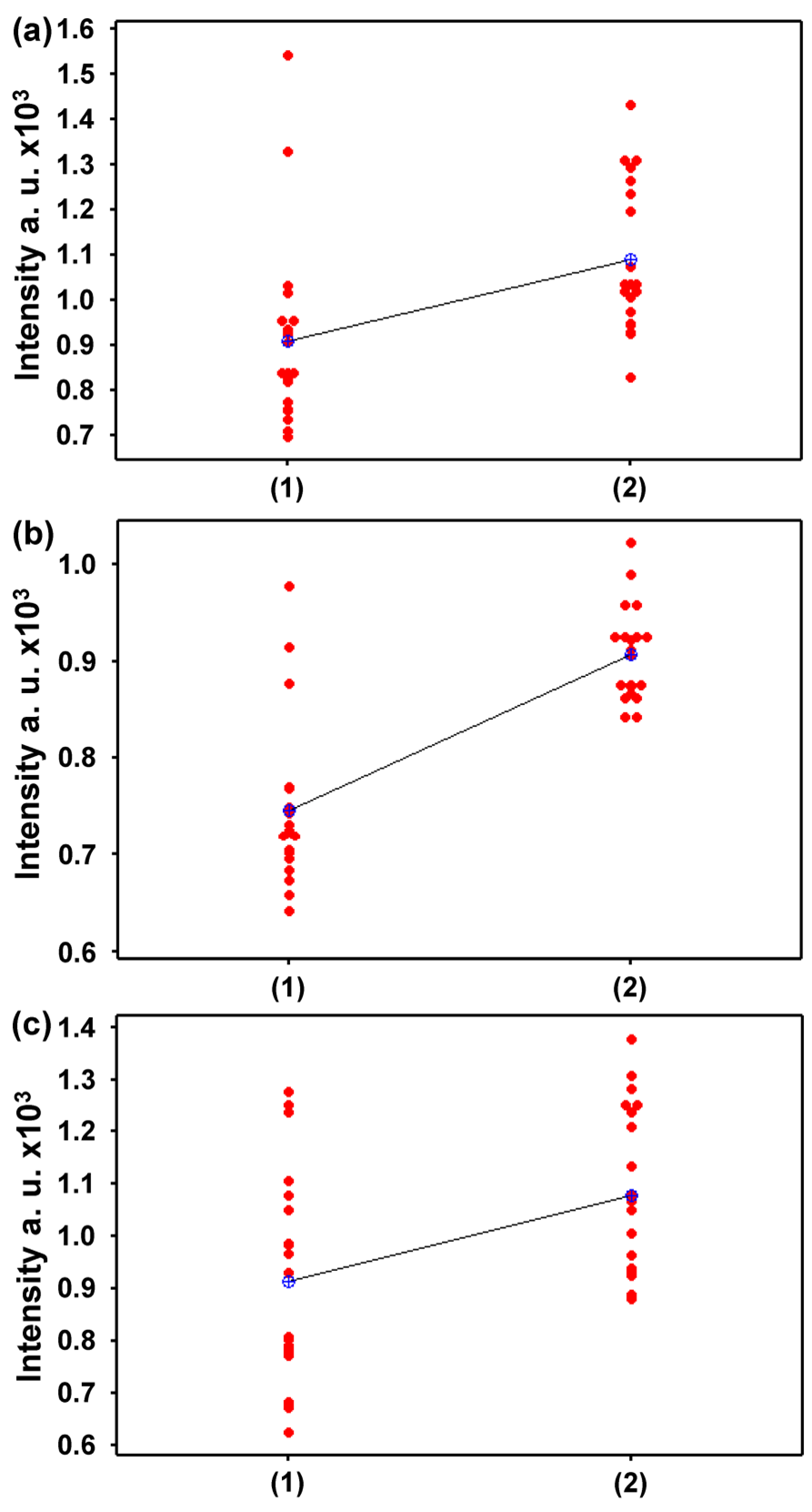

Supplement: Figure S1 — Probability distributions of SERS intensity peaks from nuclei of HEK 293T cells immobilized on (1) an Au NP array; and (2) an Au nano-dot array substrate at wavelength of (a) 835 cm−1. (b) 1006 cm−1. (c) 1155 cm−1. (TIF) [file pone.0015836.s001.tif]

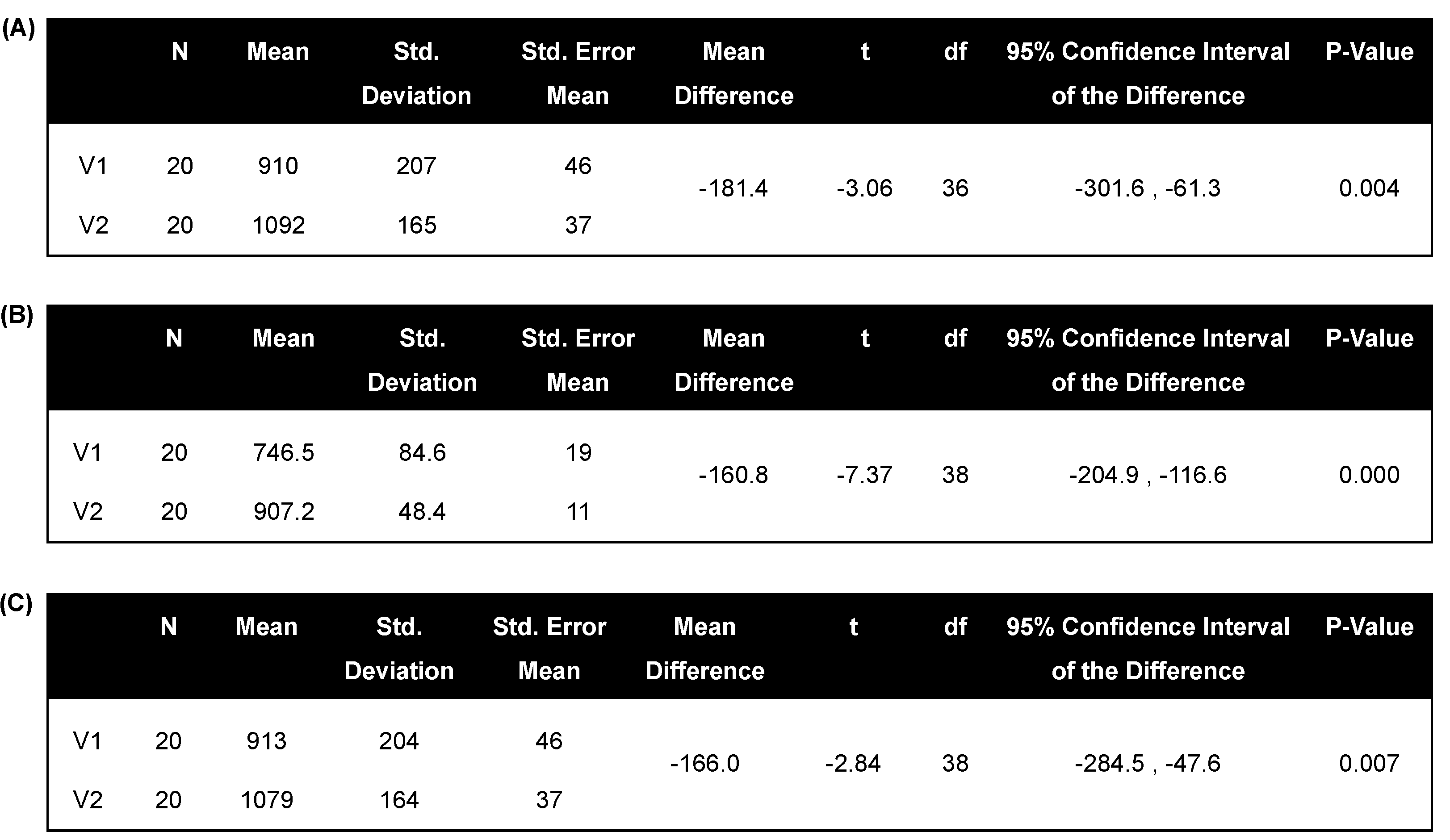

Supplement: Table S1 — Independent Student's two-sample T-test statistical analysis for comparing Raman peak intensities from HEK 293T cell nuclei immobilized on (V1) an Au NP array; and (V2) an Au nanodot array at wavelengths (a) 835 cm−1 . (b) 1006 cm−1. (c) 1155 cm−1. (TIF) [file pone.0015836.s002.tif]

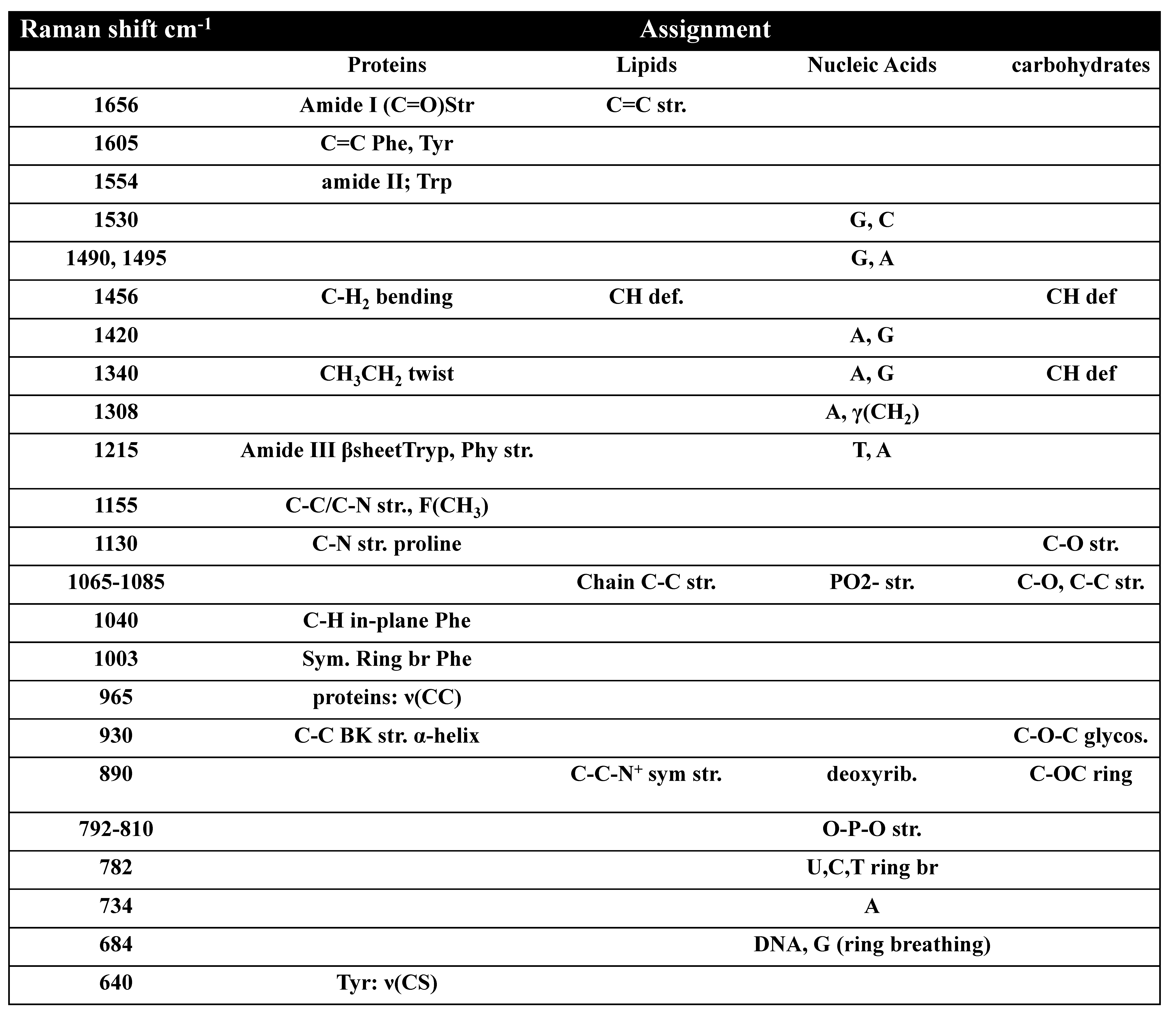

Supplement: Table S2 — Peak locations for SERS spectra of living HaLa cells. (TIF) [file pone.0015836.s003.tif]

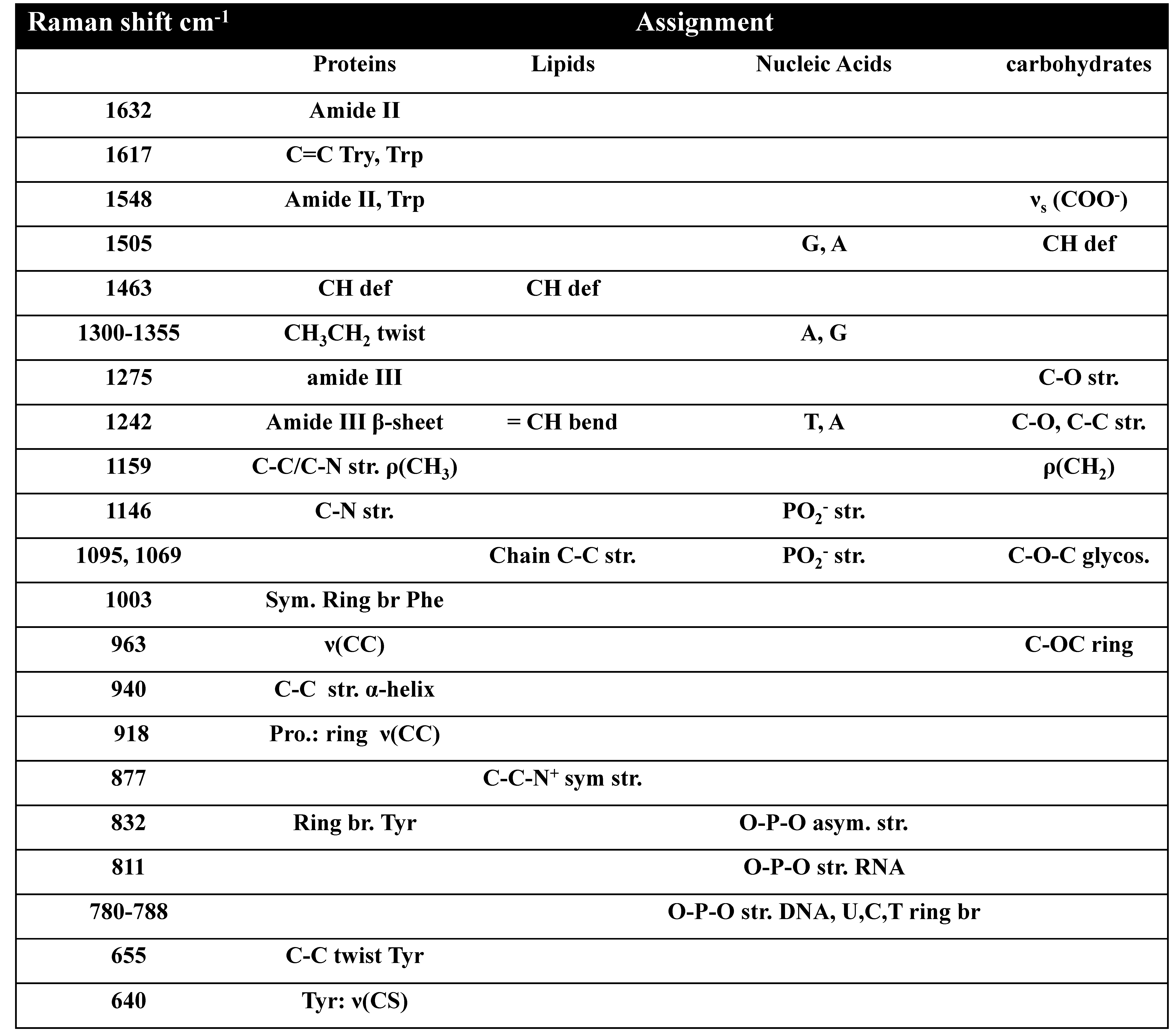

Supplement: Table S3 — Peak locations for SERS spectra of living HepG2 cells. (TIF) [file pone.0015836.s004.tif]

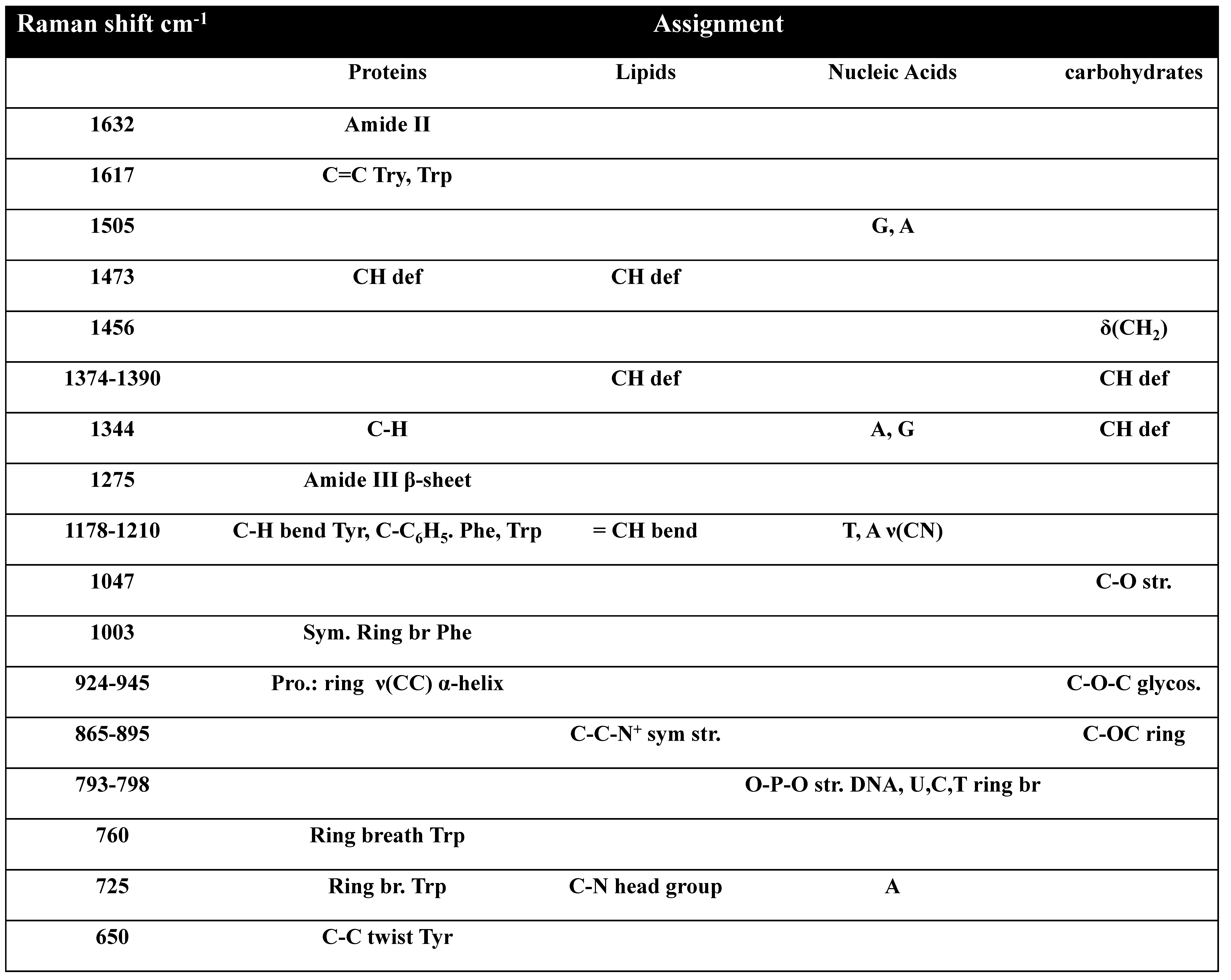

Supplement: Table S4 — Peak locations for SERS spectra of living HMCF cells. (TIF) [file pone.0015836.s005.tif]

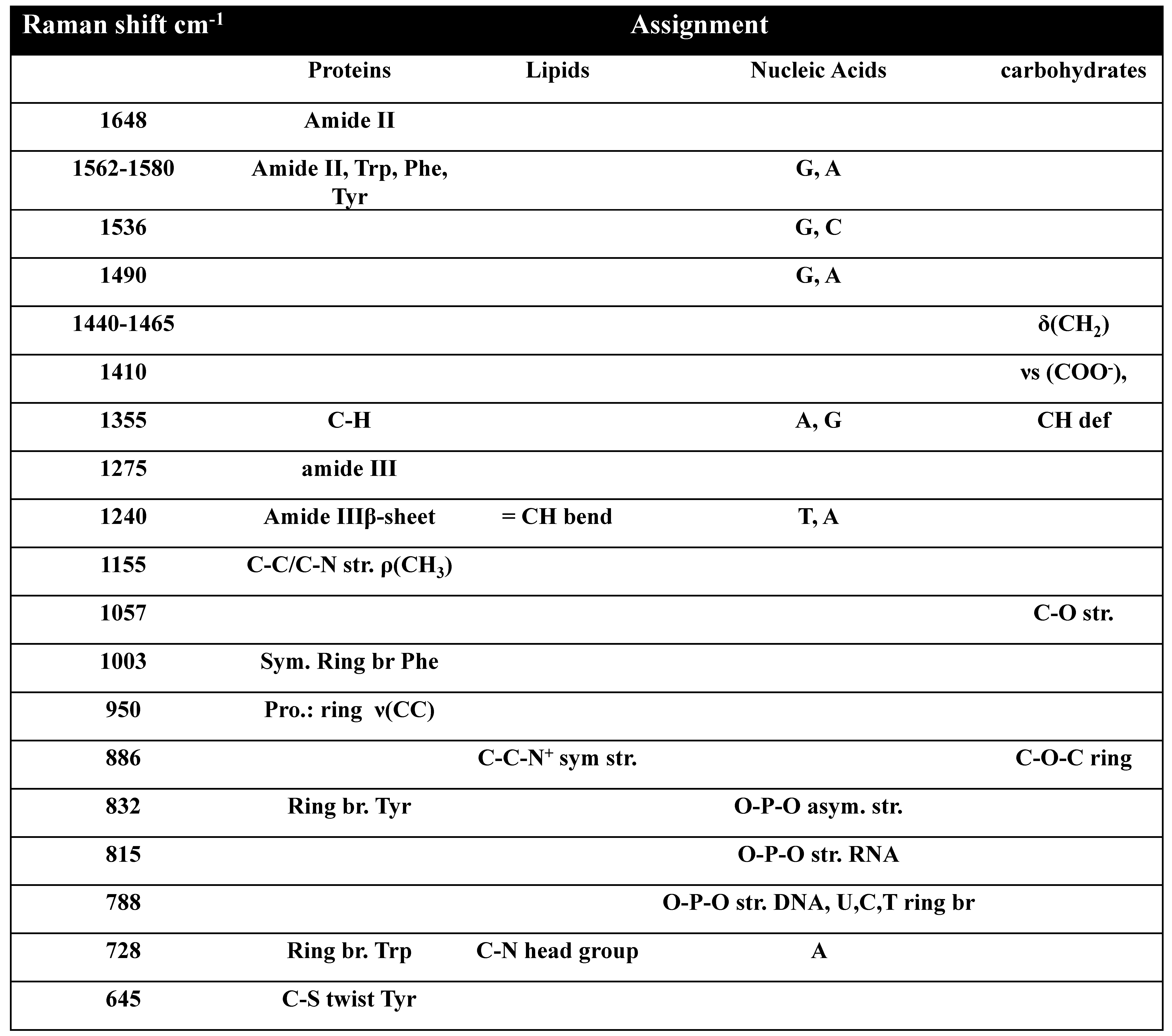

Supplement: Table S5 — Peak locations for SERS spectra of living MCF-7 cells. (TIF) [file pone.0015836.s006.tif]
